# The expression of apoptosis related genes in HK-2 cells overexpressing *PPM1K* was determined by RNA-seq analysis

**DOI:** 10.3389/fgene.2022.1004610

**Published:** 2022-11-01

**Authors:** Li Zhang, Xiaohong Sang, Yuanyuan Han, Alpati Abulitibu, Mufunayi Elken, Zhijie Mao, Shaotao Kang, Wenjun Yang, Chen Lu

**Affiliations:** ^1^ Nephrology Center of the First Affiliated Hospital of Xinjiang Medical University, Urumqi, China; ^2^ Xinjiang Clinical Research Center of Renal Replacement Therapy, Urumqi, China; ^3^ Xinjiang Branch of National Clinical Research Center for Kidney Disease, Urumqi, China; ^4^ Xinjiang Blood Purification Medical Quality Control Center, Urumqi, China; ^5^ Institute of Nephrology of Xinjiang, Urumqi, China

**Keywords:** PPM1K, human kidney-2 cell, RNA-seq, transcriptome, enrichment analysis, apoptosis

## Abstract

Chronic kidney disease (CKD) is a serious disease that endangers human health. It is reported that inhibiting renal cell apoptosis can delay the progress of CKD. Our previous study found that the mice with protein phosphatase Mg2+/Mn2+ dependent 1K (*PPM1K*) gene deletion had obvious symptoms of glomerular vascular and interstitial vascular dilatation, congestion and hemorrhage, glomerular hemorrhage and necrosis, interstitial fibrous tissue proliferation, decreased urinary creatinine clearance, and increased urinary protein level. In addition, studies have found that *PPM1K* is essential for cell survival, apoptosis and metabolism. However, no study has confirmed that *PPM1K* can inhibit renal cell apoptosis. In this study, *PPM1K* was overexpressed in human kidney-2 cells (HK-2), and the biological process of differentially expressed genes and its effect on apoptosis were comprehensively screened by RNA sequencing (RNA-seq). Through sequencing analysis, we found that there were 796 differentially expressed genes in human renal tubular epithelial cells transfected with *PPM1K* gene, of which 553 were down-regulated and 243 were up-regulated. Enrichment analysis found that differentially expressed genes may play an important role in amino acid metabolism and biosynthesis. In the GO analysis functional pathway list, we also found that multiple genes can be enriched in apoptosis related pathways, such as G0S2, GADD45A, TRIB3, VEGFA, NUPR1 and other up-regulated genes, and IL-6, MAGED1, CCL2, TP53INP1 and other down-regulated genes. Then we verified these differentially expressed genes by RT-PCR, and found that only the RT-PCR results of G0S2, VEGFA and NUPR1 were consistent with the transcriptome sequencing results. We believe that G0S2, VEGFA, NUPR1 and other genes may participate in the apoptosis process of HK-2 cells induced by *PPM1K*.In conclusion, these findings provide some data support for the study of HK-2 cell apoptosis mechanism, and also provide a scientific theoretical basis for further study of the effect of *PPM1K* on kidney disease.

## Introduction

Chronic kidney disease (CKD) has the characteristics of high prevalence, low awareness, poor prognosis and high medical costs. It is another disease seriously endangering human health after cardiovascular and cerebrovascular diseases, diabetes and malignant tumors (Hwang et al., 2012; Ene-Iordache et al., 2016). Renal tubules are the main part of the kidney and are vulnerable to various injuries, including hypoxia, proteinuria, toxins, metabolic disorders and aging. Studies have found that renal tubular cell injury drives the development of CKD. When suffering from injury reaction, renal tubular epithelial cells change and play a role as inflammatory cells and fibrotic cells, thus producing various bioactive molecules to drive interstitial inflammation and fibrosis (Liu et al., 2018). Therefore, it is necessary to explore the regulatory mechanism of renal tubular injury in the process of CDK, including tubular cell apoptosis and inflammatory transformation, in order to delay the progress of CKD.

Apoptosis is one of the important defense mechanisms of cells. It is a programmed death reaction, which is used to clear damaged cells and maintain the homeostasis of the host. It is closely related to cell replication, proliferation, immunity and cancer. Cell dysfunction and apoptosis are also key factors in the progress of chronic kidney disease (CKD) (Xiang et al., 2021). Excessive apoptosis may lead to disease. Lin Xie found that homocysteine promotes podocyte apoptosis by regulating epigenetic modifiers DNMT1 and EZH2, which is accompanied by glomerular damage, leading to the progress of CKD (Xie et al., 2021). Nan Yu found that podocytes are an important part of renal glomerular filtration barrier, and podocyte dysfunction and apoptosis are important factors leading to the progress of chronic kidney disease (CKD) (Yu et al., 2020). Izquierdo research found that abnormal blood lipids and lipid accumulation in the kidney may lead to changes in renal lipid metabolism, thus inducing endoplasmic reticulum (ER) stress and the production of reactive oxygen species (ROS), and ultimately leading to cytotoxicity, apoptosis and inflammation, which play an important role in advanced CKD (Izquierdo-Lahuerta et al., 2016). In addition, studies have shown that inhibiting renal tubular cell apoptosis can alleviate the progress of CKD. Xiang Cheng Xie found that relaxin inhibits human tubular epithelial cell apoptosis induced by Campbell acid by activating pi3k/akt signaling pathway (Xie et al., 2017); Kahori Nasu found that mint3 in epithelial cells up regulates NF- κ The anti apoptotic effect of B protects cells from apoptosis and thus inhibits fibrosis, which may be the therapeutic target of CKD (Nasu et al., 2020); Sandra Kostic found that apoptosis inducing factor (AIF) is a key factor leading to mitochondrial apoptosis, and plays an important role in apoptosis, cell function and homeostasis, and proved that AIF is a potential therapeutic target and marker of CKD progression (Kostic et al., 2020); Nan Yu confirmed that curcumin reduced podocyte injury and apoptosis by inhibiting endoplasmic reticulum stress, thereby alleviating the progress of CKD (Yu et al., 2020); The above research shows that inhibiting renal cell apoptosis is of great significance to alleviate the progress of CKD.

RNA sequencing (RNA-seq) technology is the second-generation sequencing technology with high sensitivity, high accuracy and low cost (Wang et al., 2009; Hrdlickova et al., 2017). At present, this technology is increasingly used in the analysis of diseases and drugs at the genetic level. The transcriptome sequencing data analysis using RNA-seq technology to study the expression of disease related genes mainly focuses on the analysis of differentially expressed genes, GO (Gene Ontology) function enrichment analysis and KEGG (Kyoto Encyclopedia of Genes and Genomes) pathway enrichment analysis. GO function analysis is to use the annotation results of the database to explain the functional parts of differentially expressed genes in the three categories of gene Ontology: molecular function, cellular component, and biological process (Ashburner et al., 2000). KEGG is a comprehensive database for understanding cells, organisms and ecosystems, and a database resource for practical programs of genome sequencing and other high-throughput experimental technologies generated from molecular level information, especially large molecular datasets (Kanehisa et al., 2004), which is divided into three categories: system information, genome information and chemical information. KEGG pathway is a database of system information and a collection of hand-painted metabolic pathways, including metabolism, genetic information processing, environmental information, cell process processing, biological system, human disease and drug development. At present, transcriptome sequencing technology is used in the field of kidney disease. Meeyoung Park et al. used transcriptome sequencing technology to analyze the steady-state gene expression pattern of human kidney tissue under ischemia and reperfusion conditions. Differential expression analysis showed that the metabolic pathway of cells was disordered during ischemia, and cell development, migration and immune response related pathways were out of tune during reperfusion. Cluster analysis showed that the metabolism, apoptosis and fibrosis related pathways mediated by ischemia were significantly dysregulated, while the cell growth, migration and immune response related pathways were highly dysregulated in post ischemia reperfusion. During ischemia, the expression of pro apoptotic genes and death receptors were down regulated, indicating that there was a protective mechanism against ischemic injury (Park et al., 2020). Kelly et al. Conducted transcriptome sequencing on the rat model of diabetes nephropathy to deeply study the pathogenic mechanism of diabetes nephropathy with progressive CKD. The results showed that the pathological genes of the model led to kidney inflammation, promoted apoptosis and blocked cell cycle (Kelly et al., 2013). This study can be used to develop new clinical treatment strategies.

Our previous study found that mice with protein phosphatase Mg2+/Mn2+ dependent 1K (*PPM1K*) gene deletion significantly developed glomerular and interstitial vascular dilatation, congestion and hemorrhage, glomerular hemorrhagic necrosis, interstitial fibrous tissue proliferation with inflammatory cell infiltration, significantly decreased urinary creatinine clearance, and increased urinary protein levels. We speculate that *PPM1K* may play an important protective role in the development of renal fibrosis, but its molecular mechanism is not clear. Kun Lian found that the cardiac function of diabetes mice overexpressing *PPM1k* was improved, and the myocardial infarction area and apoptosis were reduced (Lian et al., 2020). Alfonso confirmed that *PPM1K* is crucial to cell survival, apoptosis and metabolism (Oyarzabal et al., 2013). Therefore, we speculate that *PPM1K* can alleviate the progress of CKD by inhibiting renal cell apoptosis. However, no study has confirmed that *PPM1K* can inhibit renal cell apoptosis. In this study, *PPM1K* was overexpressed in human kidney-2 cells (HK-2). As the research object, the biological process of differentially expressed genes and its effect on apoptosis were comprehensively screened by RNA sequencing (RNA-seq). It is hoped that this study will bring a new way for the treatment of CKD.

## Materials and methods

### Culture of human renal tubular epithelial cells

HK-2 cells were purchased from Wuhan prosai Life Technology Co., Ltd (Wuhan, China) and inoculated into a 25 cm^2^ culture flask after resuscitation. The culture medium was DMEM medium (Gibico, Grand Island, New York, United States) containing 10% Fetal Bovine Serum (Gibico, Sandringham Ave, Thornton, NSW 2322, Australia) by mass. The culture flask was placed in a cell incubator (Thermo science, United States) with a volume fraction of 5% carbon dioxide at 37°C. Subculture when the cell fusion rate is 80%–90% under the inverted microscope.

### Construction of *PPM1K* plasmid

Hunan Youbao biological company (Hunan, China) was entrusted to construct *PPM1K* plasmid. After gene sequencing experiments, the accuracy of the sequence was proved.

### Plasmid transfection HK-2

The third generation human HK-2 is selected, and the cell density reaches 70%–80%. Two hours before transfection, the cells were cultured in serum-free medium, and 2.5 μG plasmid, add 100 μL incubate with opti MEM for 5min, and take 5 μL Lipofectamine ™ 2000(Invitrogen, Carlsbad, CA, United States) join 100 μL opti MEM, incubate for 5min and mix the above reagents. Add the above mixture to the cell culture medium, and change the fresh medium after 4–6 h.

### Western blot verifies the expression of *PPM1K*

Discard the culture medium, digest the cells with 0.25% trypsin solution (Thermo science, United States), collect the digested cells to 1.5 ml EP tube, centrifuge the supernatant and add 100 μ L Ripa (Thermo science, United States) cracking liquid, put it on ice to crack for 30min. After lysis, centrifuge for 15min (12000r/min), and take the supernatant for protein quantification. Prepare the quantitative protein solution and cook it at 100°C for 10 min. Make 10% SDS-PAGE gel (Thermo science, United States) and add 30% for each lane μG protein. Turn on the electrophoresis apparatus (Bio-Rad, United States), the upper glue voltage is 80 V (30min), the lower glue voltage is 100 V (2H), and the film is turned for 2 h (100V). After the film transfer, 5% skimmed milk was sealed for 2 h, and tbst was cleaned three times. Cut the PVDF membrane, add 3 ml of marker antibody (*PPM1K*) (1:1,000, 66008-2-IG, Proteintech) antibody and 3 ml of internal reference GAPDH (1:5,000, ATPA00013Rb, AtaGenix) antibody on the membrane, shake it overnight at 4°C, take it out on the second day, wash tbst for three times, add secondary antibody (Goat anti rabbit IgG 1:5000, Abcam), incubate at room temperature for 2 h, and clean tbst for three times. After cleaning, usie the enhanced chemiluminescence (ECL) reagent (Bio-Rad, 170506, United States) to avoid light and develop color. After the strip is clear, scan it with gel imaging system (Bio-Rad, United States), and calculate the gray value of the strip to be measured with image lab software.

### RNA extraction and sequencing

Total RNAs were extracted from HK2 cells using TRIzol Reagent (Invitrogen, cat. NO 15596026)following the methods by Chomczynski (Chomczynski and Sacchi, 1987). DNA digestion was carried out after RNA extraction by DNaseI. RNA quality was determined by examining A260/A280 with Nanodrop™ OneCspectrophotometer (Thermo Fisher Scientific Inc). RNA Integrity was confirmed by 1.5% agarose gel electrophoresis. Qualified RNAs were finally quantified by Qubit3.0 with Qubit™ RNA Broad Range Assay kit (Life Technologies, Q10210).2 μg total RNAs were used for stranded RNA sequencing library preparation using KCTM Stranded mRNA Library Prep Kit for Illumina (Catalog NO. DR08402, Wuhan Seqhealth Co., Ltd. China) following the manufacturer’s instruction. PCR products corresponding to 200–500 bps were enriched, quantified and finally sequenced on Novaseq 6000 sequencer (Illumina) with PE150 model.

### RNA-seq raw data clean and alignment

Raw reads containing more than 2-N bases were first discarded. Then adaptors and low-quality bases were trimmed from raw sequencing reads using FASTX-Toolkit (Version 0.0.13). The short reads less than 16 nt were also dropped. After that, clean reads were aligned to the GRCh38 genome by HISAT2 allowing four mismatches (Kim et al., 2015). Uniquely mapped reads were used for gene reads number counting and FPKM calculation (fragments per kilobase of transcript per million fragments mapped) (Trapnell et al., 2010).

### Differentially expressed genes (DEG) analysis

DEseq2 will model the original reads and use the scale factor to explain the difference of Library depth (Love et al., 2014). Then DEseq2 estimates the gene dispersion, and reduces these estimates to produce more accurate dispersion estimates, so as to model the reads count. Finally, the model of negative binomial distribution is fitted by DEseq2, and the hypothesis is tested by Wald test or likelihood ratio test. DEseq2 can be used to analyze the differential expression between two or more samples, and the analysis results can be used to determine whether a gene is differentially expressed by fold change (FC) and probability value (*p* value). The *p* value for correction <0.05 and fold change>2 or <0.5 were set as the cut-off criteria for identifying DEGs.

### Functional enrichment analysis

To sort out functional categories of DEGs, GO terms and KEGG pathways were identified using KOBAS 2.0 server (Chen et al., 2011). Hyper geometric test and Benjamini–Hochberg FDR controlling procedure were used to define the enrichment of each term.

### RT-qPCR validation of DEGs

In order to clarify the reliability of DEGs in overexpressing *PPM1K* in HK-2 cells, RT-qPCR was performed on some of the up-regulated and down-regulated DEGs. The RNA samples used for RT-qPCR are the same as those used for RNA sequences. The PCR conditions were denaturation at 95°C for 10 min, then denaturation at 95°C for 15 s, annealing and extension at 60°C for 1 min, a total of 40 cycles. Then extend at 60°C for 1 min, and store the product at 4°C. All samples were amplified by PCR three times.

### Statistical analysis

The experimental data are expressed by mean ± SD, and statistical analysis is carried out by SPSS v22.0 software (IBM SPSS statistics for Windows). Using one-way ANOVA Tukey’s HSD test, *p* < 0.01, it is considered that there is a statistical difference.

## Result

### Morphological observation of HK-2 cells

HK-2 cells grow adherently, with different sizes, irregular shapes, circular and spindle shapes. They proliferate in a whirlpool. The cells in the center of the whirlpool are dense, and one to two nucleoli can be seen in the cells. Only a small number of floating cells and cell fragments are left in the culture medium; After transfection with *PPM1K*, the cell morphology changed little, such as round, spindle, *etc.*, the cell proliferation rate did not change, and only a small number of floating cells and cell fragments remained in the culture medium.

### The overexpression of *PPM1K* was verified by western blot

**FIGURE 1:**
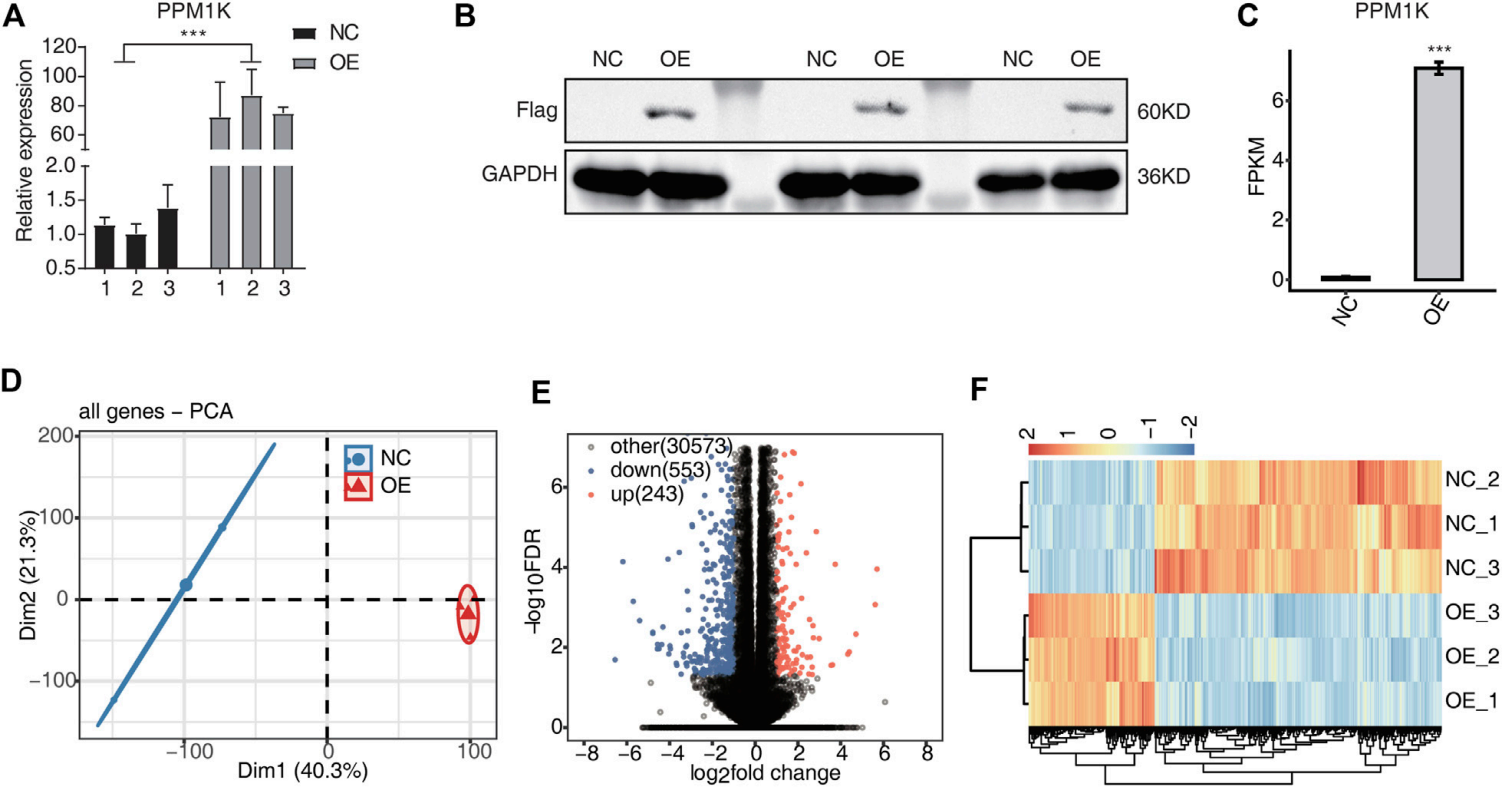
*PPM1K*-OE affect gene expression profiles of HK2 cells **(A)** The histogram showed the RT-qPCR results of control and treatment samples. Error bars represent mean ± SEM. *** *p*-value < 0.001. **(B)** The result of western blot experiment showed that the *PPM1K* over expression was successful **(C)** Bar plot showing the expression pattern and statistical difference of DEGs for *PPM1K*. Error bars represent mean ± SEM. ***p-value < 0.001. **(D)** PCA base on FPKM value of all detected genes *PPM1K* overexpression. The ellipse for each group is the confidence ellipse **(E)** Volcano plot showing all differentially expressed genes (DEGs) between overexpression (OE) and Ctrl samples. **(F)** Hierarchical clustering heat map showing expression levels of all DEGs.

Western blot showed that the expression of *PPM1K* protein existed in HK-2 cells transfected with *PPM1K* gene (Figure 1B).

### PCR experiment verifies the overexpression of *PPM1K*

PCR results showed that *PPM1K* gene was successfully transfected into HK-2 cells, and the transfection efficiency was 78.2% (*p* < 0.01). The transfection efficiency is high, and *PPM1K* is stably expressed, which can be used in subsequent experiments (Figure 1A).

### Transcriptome sequencing quality evaluation

Transcriptome sequencing results show that the percentage of quality value 20 (Q20) is 100%, the percentage of quality value 30 (q30) is more than 99%, and the proportion of GC content is 50%–50.5% (Table 1). The overall sequencing quality is good, and subsequent analysis can be carried out. According to the characteristics of different genomes, compare the effective sequencing data (clean reads) to the reference genome (Table 2). Statistics of the distribution of reads with unique location on the genome in each region (Table 3).

**TABLE 1:** Valid sequence table.

SampleID	Raw	Clean	Clean Per(%)	Raw base(G)	Clean base(G)	Base Per(%)	Q20 (%)	Q30 (%)	GC (%)	DUP(%)
NC_1	81569682	79951252	98.02	12.24	11.62	94.93	100	99.15	50.5	30.9
NC_2	84783386	83243760	98.18	12.72	12.07	94.89	100	99.2	50	32.7
NC_3	85184602	83679342	98.23	12.78	12.16	95.15	100	99.2	50.5	30
OE_PPM1K _1	87842902	86357578	98.31	13.18	12.53	95.07	100	99.2	50.5	29.5
OE_PPM1K _2	92718702	91075144	98.23	13.91	13.26	95.33	100	99.15	50.5	28.8
OE_ PPM1K _3	83827288	82362784	98.25	12.57	11.97	95.23	100	99.2	50.5	31.1

**TABLE 2:** Mapping of clean reads on the reference genome.

Sample	NC_1	NC_2	NC_3	OE_PPM1K_1	OE_PPM1K_2	OE_PPM1K_3
Total reads	79951252	83243760	83679342	86357578	91075144	82362784
Total mapped	75935773 (94.98%)	78651556 (94.48%)	79288743 (94.75%)	81692131 (94.60%)	86169279 (94.61%)	77942656 (94.63%)
Total Uniquely mapped	72136329 (95.00%)	74747149 (95.04%)	75255465 (94.91%)	77474949 (94.84%)	81695047 (94.81%)	74129880 (95.11%)
Total Multiple mapped	3799444 (5.00%)	3904407 (4.96%)	4033278 (5.09%)	4217182 (5.16%)	4474232 (5.19%)	3812776 (4.89%)
Total Pairs	39975626	41621880	41839671	43178789	45537572	41181392
Total Uniquely Concordant Pairs	34795095 (87.04%)	36016307 (86.53%)	36312355 (86.79%)	37343261 (86.49%)	39443495 (86.62%)	35760567 (86.84%)
Splice reads	40587436 (56.26%)	39704102 (53.12%)	41792107 (55.53%)	44111549 (56.94%)	47131803 (57.69%)	42526915 (57.37%)
Nonsplice reads	31548893 (43.74%)	35043047 (46.88%)	33463358 (44.47%)	33363400 (43.06%)	34563244 (42.31%)	31602965 (42.63%)

**TABLE 3:** Reads distribution across reference Genomic Regions.

Sample	5′UTR	3′UTR	CDS	Nc_exon	Introns	Intergenic	Antisense
NC_1	4179928 (5.79%)	7730910 (10.72%)	55093867 (76.37%)	2015322 (2.79%)	1997313 (2.77%)	484181 (0.67%)	634803 (0.88%)
NC_2	4149816 (5.55%)	7941809 (10.62%)	54569187 (73.01%)	2235797 (2.99%)	2977100 (3.98%)	1403033 (1.88%)	1470404 (1.97%)
NC_3	4248847 (5.65%)	8330963 (11.07%)	57092862 (75.87%)	2192660 (2.91%)	2112622 (2.81%)	554408 (0.74%)	723099 (0.96%)
OE_PPM1K_1	4841226 (6.25%)	8410511 (10.86%)	59172838 (76.38%)	2206522 (2.85%)	1879153 (2.43%)	416448 (0.54%)	548247 (0.71%)
OE_PPM1K_2	4997566 (6.12%)	9063648 (11.09%)	62842610 (76.92%)	2135777 (2.61%)	1680215 (2.06%)	411526 (0.50%)	563701 (0.69%)
OE_PPM1K_3	4756867 (6.42%)	7972704 (10.76%)	56693912 (76.48%)	1993208 (2.69%)	1801478 (2.43%)	398243 (0.54%)	513463 (0.69%)

### The effect of overexpression of *PPM1K* gene on the transcriptome of HK-2 cells

RNA-seq results showed that 31369 genes were co expressed in overexpression group (OE) and control group (NC) cells, and 796 genes were specifically expressed, of which 243 genes were up-regulated and 553 genes were down regulated (Figure 1E). The down regulated genes were significantly more than the down regulated genes (FC ≥ 2 or ≤0.5, FDR <0.05). The heat map shows that NC group and OE group are different, and the two biological replication are highly correlated (Figure 1F).

**FIGURE 2:**
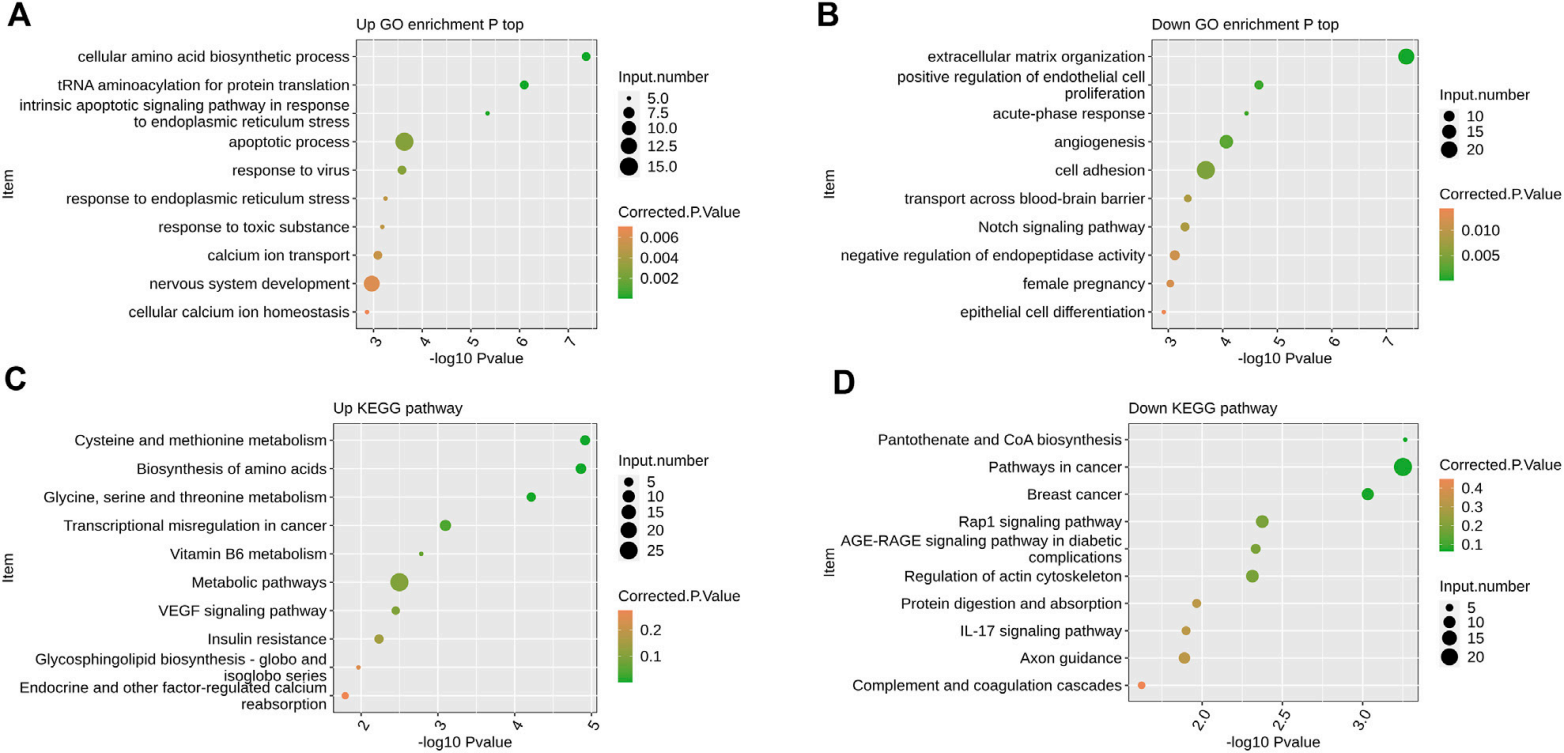
The effect of overexpression of *PPM1K* gene on the transcriptome of HK-2 cells **(A)** Scatter plot exhibiting the most enriched GO biological process results of the up-regulated DEGs **(B)** Scatter plot exhibiting the most enriched GO biological process results of the down-regulated DEGs. **(C)** Scatter plot exhibiting the most enriched KEGG pathway results of the up-regulated DEGs. **(D)** Scatter plot exhibiting the most enriched KEGG pathway results of the down-regulated DEGs.

By extracting the up-regulated epitope genes after *PPM1K* overexpression, GO analysis showed that the up-regulated genes were significantly enriched in the cellular amino acid biosynthetic process, tRNA aminoacylation for protein translation, intrinsic apoptotic signaling pathway in response to endoplasmic reticulum stress, apoptotic process, response to virus, response to endoplasmic reticulum stress, response to toxic substance, calcium ion transport, nervous system development, cellular calcium ion homeostasis and other biological processes (Figure 2A).

GO analysis showed that the down-regulated genes were significantly enriched in extracellular matrix organization, positive regulation of endothelial cell proliferation, acute−phase response, angiogenesis, cell adhesion, transport across blood−brain barrier, Notch signaling pathway, negative regulation of endopeptidase activity, female pregnancy, epithelial cell differentiation and other biological processes (Figure 2B).

KEGG analysis showed that the up-regulated genes were significantly enriched in biological processes such as cysteine and methionine metabolism, biosynthesis of amino acids, glycine, serine and threonine metabolism, transcriptional misregulation in cancer, vitamin B6 metabolism, metabolic pathways, VEGF signaling pathway, insulin resistance, glycosphingolipid biosynthesis − globo and isoglobo series, endocrine and other factor−regulated calcium reabsorption and so on (Figure 2C).

KEGG analysis showed that the down-regulated genes were significantly enriched in biological processes such as pantothenate and CoA biosynthesis, pathways in cancer, breast cancer, Rap1 signaling pathway, AGE−RAGE signaling pathway in diabetic complications, regulation of actin cytoskeleton, protein digestion and absorption, IL−17 signaling pathway, axon guidance, complement and coagulation cascades (Figure 2D).

### *PPM1K* regulated expression of apoptosis associated genes in HK2 cells

**FIGURE 3:**
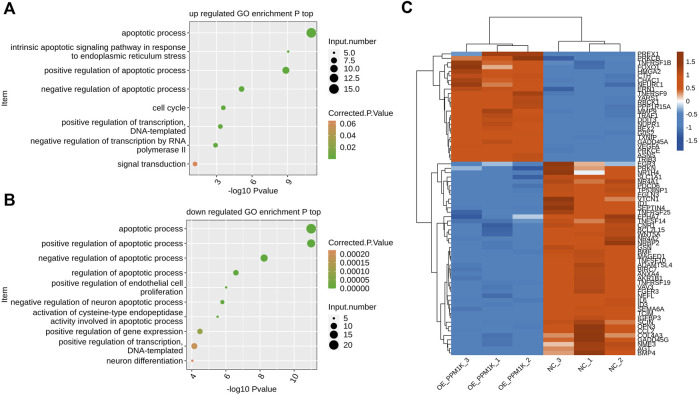
*PPM1K* regulated expression of apoptosis associated genes in HK2 cells **(A)** Scatter plot exhibiting the most enriched GO biological process results of the all up-regulated genes of apoptosis-related (top) **(B)** Scatter plot exhibiting the most enriched GO biological process results of the all down-regulated genes of apoptosis-related (bottom). **(C)** Hierarchical clustering heat map showing expression levels of all apoptosis pathway genes.

In the list of functional pathways by GO analysis, we also found that many genes can be enriched in biological processes related to apoptosis, and the up-regulated genes are significantly enriched in biological processes such as apoptotic process, intrinsic apoptotic signaling pathway in response to endoplasmic reticulum stress, positive regulation of apoptotic process, negative regulation of apoptotic process, cell cycle, positive regulation of transcription, DNA−templated, negative regulation of transcription by RNA polymerase II, signal transduction and so on (Figure 3A).

The down-regulated genes are significantly enriched in biological processes such as apoptotic process, positive regulation of apoptotic process, negative regulation of apoptotic process, regulation of apoptotic process, positive regulation of endothelial cell proliferation, negative regulation of neuron apoptotic process, activation of cysteine−type endopeptidase activity involved in apoptotic process, positive regulation of gene expression, positive regulation of transcription DNA−templated, neuron differentiation (Figure 3B).

### RT-PCR verifies the reliability of sequencing

**FIGURE 4:**
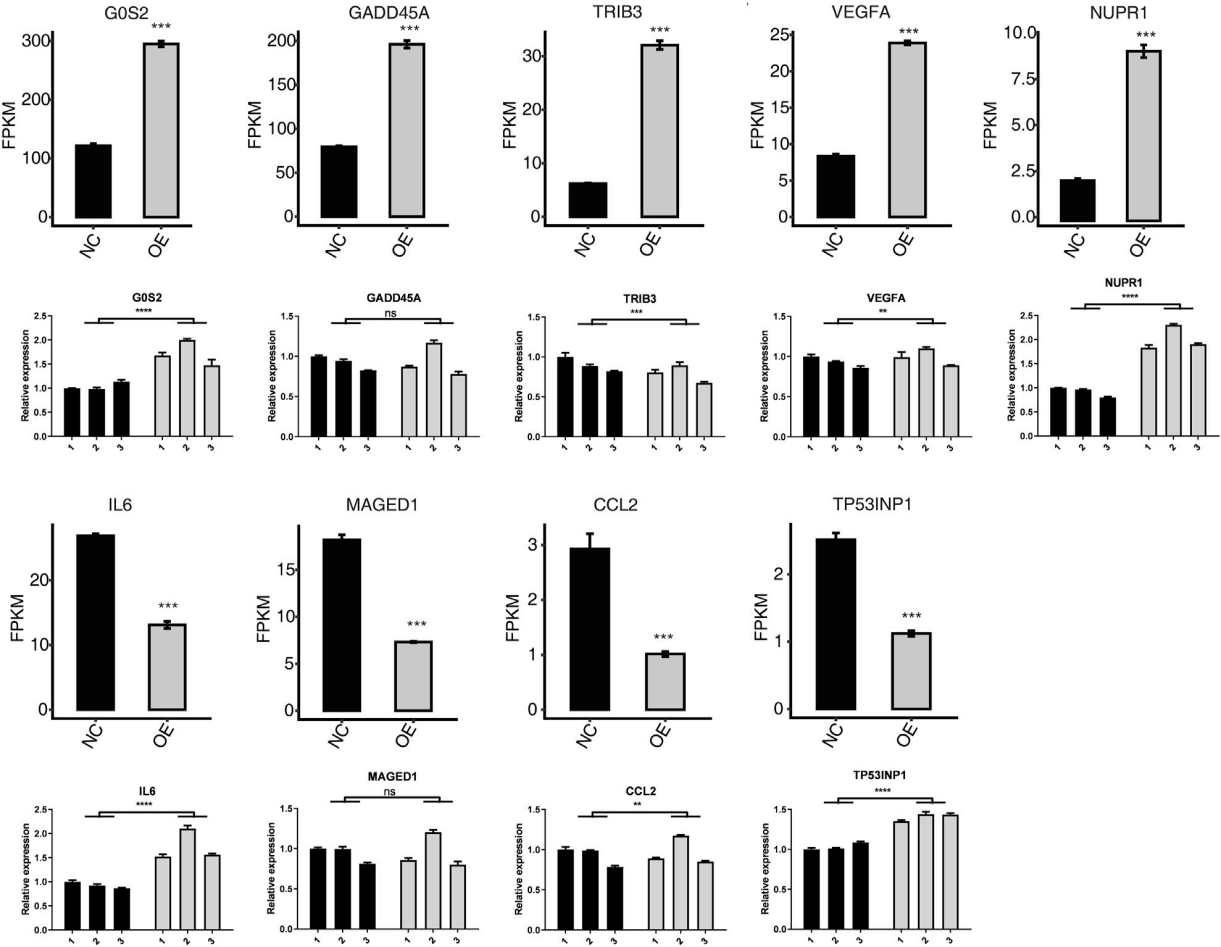
Related expression level (FPKM) and RT-qPCR measurement of apoptotic-related DEGs. ****p-value < 0.0001. ***p-value < 0.001. **p-value < 0.01.

In order to verify the expression of apoptosis gene in HK-2 cells overexpressing *PPM1K*, we used RT-PCR to verify. In the GO functional pathway list, we performed RT-PCR verification on the up-regulated genes such as G0S2, GADD45A, TRIB3, VEGFA and NUPR1, and RT-PCR verification on the down-regulated genes such as IL-6, MAGED1, CCL2 and TP53INP1. The results showed that the RT-PCR expression of G0S2, GADD45A, VEGFA and NUPR1 genes increased in HK-2 cells overexpressing *PPM1K* gene, which was consistent with the transcriptome sequencing results. The RT-PCR results of G0S2, VEGFA and NUPR1 were statistically significant; The RT-PCR expression of TRIB3 gene decreased, which was statistically significant, but was inconsistent with the transcriptome sequencing results. In HK-2 cells overexpressing *PPM1K* gene, the RT-PCR expression of IL-6, MAGED1, CCL2 and TP53INP1 genes increased, and the RT-PCR results of IL-6, CCL2 and TP53INP1 were statistically significant; The RT-PCR results of MAGED1 were not statistically significant, but the RT-PCR results of IL-6, MAGED1, CCL2 and TP53INP1 were inconsistent with the transcriptome sequencing results (Figure 4).

## Discuss

The single nucleotide polymorphism (SNP) of *PPM1K* leading to protein function defects is associated with many human diseases, such as cardiovascular disease, maple diabetes disease (MSUD), type 2 diabetes and nervous system diseases (Rostaminasab et al., 2018). However, the research on the mechanism of the important role of *PPM1K* in kidney disease is still limited. In this study, we overexpressed *PPM1K* in HK-2 cells, trying to analyze the biological process of differentially expressed genes and their effects on apoptosis through transcriptome.

Apoptosis occurs through two pathways, endogenous and exogenous apoptosis, both of which induce caspase cascade (Kroemer et al., 2007). Apoptosis is considered to be an important process of cell death (Elmore, 2007). In the GO analysis functional pathway list, we also found that multiple genes can be enriched in apoptosis related pathways, such as G0S2, GADD45A, TRIB3, VEGFA, NUPR1 and other up-regulated genes, and IL-6, MAGED1, CCL2, TP53INP1 and other down-regulated genes. Then we verified these differentially expressed genes by RT-PCR, and found that only the RT-PCR results of G0S2, VEGFA and NUPR1 were consistent with the transcriptome sequencing results, while the RT-PCR results of TRIB3, IL-6, MAGED1, CCL2 and TP53INP1 were inconsistent with the transcriptome sequencing results. Therefore, we believe that G0S2, VEGFA, NUPR1 and other genes may participate in the apoptosis process of HK-2 cells induced by *PPM1K*.

G0S2 is a small protein with a length of 103 residues, which is involved in a variety of cellular processes (Russell and Forsdyke, 1991). In lipolysis, G0S2 interacts specifically with triglyceride lipase to inhibit its activity, resulting in down-regulation of lipolysis. Studies have found that the function of G0S2 is to act directly with key proteins. In a similar way, G0S2 is also involved in the regulation of apoptosis, cell proliferation and oxidative phosphorylation (Páez-Pérez et al., 2020). In mitochondria, G0S2 interacts specifically with Bcl-2 to prevent its interaction with Bax. In this way, G0S2 inhibits the formation of anti apoptotic heterodimer complex bcl-2/bax, which is conducive to apoptosis (Welch et al., 2009).

Nucleoprotein-1 (NUPR1) was initially found to be expressed as a small protein in rats and as a new gene activated during the acute phase of induced pancreatitis and pancreatic development (Mallo et al., 1997). NUPR1 regulates cell injury and death in different forms, depending on the cellular environment and the type of stress induction. NUPR1 is involved in D9 tetrahydrocannabinol (THC) - induced cancer cell death through downstream targeted death inducing telomere repeat binding factor 3 (TRB3) protein (Martin et al., 2021). NUPR1 knockout can regulate autophagy by interfering with FoxO3 and promoting BNIP3 transcription to control autophagy, which is a stress-dependent self-defense mechanism that helps cells eliminate toxic microenvironment (Kong et al., 2010). It is reported that NUPR1 silences PI3K/AKT mammalian targets through the rapamycin (mTOR) pathway, inhibits autophagy activity of multiple myeloma (mm) cells and induces autophagy mediated apoptosis (Li et al., 2020).

Vascular endothelial growth factor (VEGF) is an endothelial cell specific protein, which can regulate the activity of endothelial cells, promote the formation of new blood vessels, and chemotactic vascular endothelial cells and monocytes, and mediate angiogenesis (Almawi and Saldanha., 2013). Vascular endothelial growth factor A (VEGFA) is a highly effective angiogenesis factor, which can increase vascular permeability and prevent apoptosis. Ting Liu found that metformin can protect kidney by activating Hif-2 α- VEGF signal pathway can reduce the proteinuria of hypertensive rats (Liu et al., 2020). Some studies have found that VEGFA signal can maintain the activity of glomerular endothelial cells, but when the concentration of VEGFA is too high, it may lead to endothelial cell damage (Brosius and Coward, 2014). In the disease of endothelial hyperplasia, excessive release of VEGFA leads to endothelial cell proliferation and swelling, but too little release of VEGFA will lead to endothelial damage and apoptosis, resulting in glomerulosclerosis (Sivaskandarajah et al., 2012). Fan found that inhibiting VEGFA can inhibit cell proliferation, promote cell apoptosis, and thus inhibit cell migration and invasion (Zhengyan et al., 2016). Therefore, in order to maintain the structure and function of the glomerulus, it is necessary to regulate VEGFA signal transduction and regulate the concentration of VEGFA. In our study, although we found that VEGFA may participate in the apoptosis process of HK-2 cells induced by *PPM1K*, we also speculated that HK-2 cells expressing *PPM1K* may activate VEGFA signals. In order to verify this assumption, we will conduct more in-depth research.

Gadd45a gene is a member of the Gadd45 family, which is involved in the regulation of cell cycle, immunity and apoptosis (Fang et al., 2018). Under the induction of hypertonic pressure, renal medulla cells secrete Gadd45 protein. Hypertonic stress beyond a certain threshold will strongly induce apoptosis (Mak and Kultz, 2004). TRIB3 is a pseudokinase in mammals, which can control cell proliferation, apoptosis, migration and invasion (Ohoka et al., 2005; Salazar et al., 2009; Quan et al., 2016). Huo up-regulated the expression of TRIB3 level in mice. The results showed that there was obvious ER stress reaction in the kidney, liver and lungs of mice, and apoptosis was found in the lungs and kidneys of mice (Lingling et al., 2015). Although our sequencing results show that GADD45A and TRIB3 are involved in the process of cell apoptosis, the PCR results do not support it. Later, we will further verify it through protein detection and other experiments.

IL-6 is a typical cytokine, which also has pro-inflammatory and anti-inflammatory effects (Ju et al., 2016). IL-6 can activate JAK-STAT signal pathway through membrane-bound IL-6 receptor (classical pathway) or soluble IL-6 receptor (cross signal) (Hebenstreit et al., 2005), IL-6 connected to the receptor can start cascade reaction through JAK activation, activated JAK kinase phosphorylation induces dimer formation through STAT3 phosphorylation (Rose-John., 2012; Brooks et al., 2014), and this signal pathway promotes cell proliferation, differentiation, migration and apoptosis (Rawlings and Harrison, 2004). Some studies have found that IL-6 can play an anti apoptotic role by regulating the expression of apoptosis related proteins such as Bcl-2, Bax, etc (Meng et al., 2006; Isomoto, 2009). MAGE-D1 is a member of the type II melanoma associated antigen (MAGE) family. MAGE-D1 plays an important role in cell cycle, cell differentiation and apoptosis (Zhang et al., 2016). MAGE-D1 has long been considered as an apoptosis promoting gene. MAGE-D1 is involved in the apoptosis of gastric cancer cells, adrenal pheochromocytoma, Hela cells and other cancer cells (Kendall et al., 2005; Salehi et al., 2006; Cheng et al., 2008). Recent studies have found that MAGE-D1 may play an anti apoptotic role. Kumar et al. proposed that MAGE-D1 can inhibit the anoikis of lung cancer and breast cancer cells, and promote the anoikis sensitivity (Kumar et al., 2011). Yang et al. found that MAGE-D1 can promote the proliferation of esophageal cancer cells, so they suspected that MAGE-D1 can inhibit the apoptosis of esophageal cancer cells. However, they did not find the difference in apoptosis between the esophageal cancer cells with MAGE-D1 knocked out and the control group (Yang et al., 2014), and other researchers also found that MAGE-D1 has no effect on apoptosis (Wen et al., 2004; Xue et al., 2005; Du et al., 2009). We believe that the effect of MAGE-D1 on apoptosis is a complex process, which needs a lot of research to confirm. C-C Motif chemokine ligand 2 (CCL2) is a member of the G protein coupled receptor family (Kuang et al., 1996; Bose and Cho, 2013). CCL2 can express chemokine C-C motif receptor 2 (CCR2) chemotactic cells, making monocytes, T lymphocytes and natural killer cells migrate and infiltrate into the inflammatory region (Semple et al., 2010). Fan found that angiopoietin-1 can reduce the expression of chemokine C-C motif ligand 2 (CCL2) in endothelial cells of fibrotic kidney, thereby inhibiting macrophage migration and reducing cell apoptosis. Nucleoprotein 1 (TP53INP1) induced by tumor protein 53 is a p53 inducible gene that encodes two protein subtypes and regulates p53 biological activity (Jiang et al., 2006). Overexpression of TP53INP1 induces cell cycle arrest in G1 phase and enhances p53 mediated apoptosis (Tomasini et al., 2003). Many studies have shown that TP53INP1 is the target gene of many miRNAs(Ma et al., 2010; Jiang, 2011; Cai et al., 2017; Dan et al., 2018), including miR-221, miR-30a and mir-205 (Chen et al., 2016; Wang et al., 2016; Xu et al., 2016). In addition, TP53INP1 is a regulator of autophagy, which may interact with autophagy related molecules, including light chain three and autophagy related protein eight family proteins, indicating that it can not only regulate, but also promote autophagy (Seillier et al., 2012; Saadi et al., 2015). In our study, we sequenced the transcriptome of HK-2 cells overexpressing *PPM1K* and found that down-regulated genes such as IL-6, MAGED1, CCL2 and TP53INP1 were enriched in apoptosis related pathways. However, we did not find any reliable basis in the subsequent PCR validation, and we will conduct more in-depth research later.

To sum up, based on RNA seq and bioinformatics analysis, this study identified and screened differentially expressed genes overexpressing *PPM1K* in HK-2 cells. Through GO and KEGG analysis, combined with PCR validation, it was further screened that G0S2, VEGFA and NUPR1 might participate in the apoptosis process of HK-2 cells induced by *PPM1K*. This study provides some data support for the study of the mechanism of HK-2 cell apoptosis, and also provides a scientific theoretical basis for the further study of the impact of *PPM1K* on kidney disease.

## Data Availability

The datasets presented in this study can be found in online repositories. The names of the repository/repositories and accession number(s) can be found below: https://www.ncbi.nlm.nih.gov/geo; GSE212681.
